# Iodine for the Dropsy, after Scarlet Fever

**Published:** 1842-10-08

**Authors:** 


*Iodine for the Dropsy after Scarlet Fever.*—Mr. Copeman, of Coltishall, writes in the Medical Gazette, in reference to dropsy after scarlet fever:—

"From having observed its power of strengthening the constitution, with, at the same time, a tendency to prevent inflammation and to increase absorption, I was induced to make a trial of iodine. I prescribed it in the form of solution recommended by Lugol, viz.: ℞. Iodin. ℈j.; Iodid. Potass. ℈ij.; Aquæ ℥vij. M. ft. Solutio Iodin. concentr.

Of this solution I ordered from 5 to 10 drops for children, and from 10 to 20 or 25 to adults, three times a day in water. In the first case in which it was used it rapidly effected a cure: in consequence of which I prescribed it in every succeeding case that presented itself, and with the same complete success."

